# Case Report: Septic arthritis in children caused by *Streptococcus pyogenes–*rational use of antibiotics

**DOI:** 10.3389/fcimb.2022.1117217

**Published:** 2023-01-18

**Authors:** Dingle Yu, Waiwai Gao, Danchun Guo, Qinghua Lu, Yunsheng Chen, Yuejie Zheng, Wenjian Wang, Yonghong Yang

**Affiliations:** ^1^ Department of Respiratory Medicine, Shenzhen Children′s Hospital, Shenzhen, Guangdong, China; ^2^ Microbiology Laboratory, National Center for Children’s Health, Beijing Pediatric Research Institute, Beijing Children’s Hospital, Capital Medical University, Beijing, China

**Keywords:** *Streptococcus pyogenes*, child, septic arthritis, antibiotics, resistance

## Abstract

To investigate the clinical characteristics and treatment of septic arthritis caused by *Streptococcus pyogenes*(*S. pyogenes*) in children, we retrospectively analyzed the clinical data, laboratory results, treatments and outcomes of three pediatric cases of septic arthritis caused by *S. pyogenes* occurring from 2016–2018. The three cases of septic arthritis included 1 boy and 2 girls, aged from 2–7 years. Two patients experienced fever, and in all three cases, the affected joints showed redness, swelling, an increased local skin temperature, tenderness and restricted limb movement. At the first visit, all three cases showed a significantly increased white blood cell count [(27.68–32.02)×10^9^/mL] and a significantly increased erythrocyte sedimentation rate (113–134 mm/h). The C-reactive protein level was significantly increased in two cases (67 mg/L, 147.7 mg/L) and normal in one case. The procalcitonin level was normal in 1 case, elevated in 1 case, and undetected in 1 case. *S. pyogenes* isolated from cases 1 and 2 were *emm*1/ST28 and from case 3 was *emm*12/ST36. All patients were treated by abscess incision and drainage, and *S. pyogenes* was cultured in the abscess puncture fluid. All patients were treated with intravenous antibiotics after admission, and all patients were cured and discharged. The patients were followed up for 2 months, and their condition was improved and stable. No sequelae such as heart and kidney damage were detected. In conclusion, for children with septic arthritis, early diagnosis and timely treatment with incision and drainage followed by culture of the abscess puncture fluid are important. Once *S. pyogenes* infection is confirmed, β-lactam antibiotics provide effective treatment, avoiding use of broad-spectrum antibiotics.

## Introduction

Group A *Streptococcus* (GAS, also known as *Streptococcus pyogenes*), is an important pathogen that most commonly colonizes the upper respiratory tract and skin epithelium (Rouchon et al., 2017). Its pathogenicity corresponds to a broad spectrum of conditions, including pharyngitis, impetigo, scarlet fever, nephritis, rheumatic fever, rheumatic heart disease, and many other diseases (Chaudhary et al., 2018) and can also lead to severe invasive infections such as sepsis, necrotizing fasciitis, and toxic shock syndrome, which are associated with high morbidity, mortality, and disability rates (McMillan et al., 2007). The pathogenic bacteria most commonly found to be responsible for septic arthritis are *Staphylococcus aureus* (Jin et al., 2015), *Haemophilus influenzae* (Zhang et al., 2019), and *Streptococcus agalactiae* (Dhekane et al., 2020), and although less common, septic arthritis caused by *S. pyogenes* infection has been reported. In the present case series, we retrospectively analyzed the clinical data of children treated for septic arthritis due to *S. pyogenes* infection in our hospital to provide a basis for the diagnosis and treatment of septic arthritis cases due to *S. pyogenes* infection.

## Data and methods

### General data

Three cases with a clinical diagnosis of septic arthritis for which pus culture in the microbiology laboratory identified *Streptococcus pyogenes* between May 2016 and January 2018 were selected as the study subjects. The diagnosis of septic arthritis was made with reference to the literature criteria (Xu et al., 2016), which include: (1) signs and symptoms of infection, such as fever, local pain, swelling, and limitation of activity; (2) imaging examinations (such as ultrasound, computed tomography [CT] or magnetic resonance imaging [MRI]) suggesting local soft tissue swelling and joint cavity effusion; (3) X-ray examination suggesting joint dislocation or subluxation or local bone destruction; (4) significant elevation of inflammatory indexes such as white blood cell (WBC) count, C-reactive protein (CRP) level, and erythrocyte sedimentation rate (ESR); and (5) extraction of pus by puncture of the joint cavity. The diagnosis of septic arthritis is made only when three or more of the above criteria are met.

### Methods

This retrospective study analyzed the clinical characteristics, laboratory results and pathological findings for three pediatric cases of septic arthritis, and the patients were followed up by telephone for more than 2 months. For bacterial culture detection, pus specimens were inoculated in 5% defibrinated sheep blood culture dishes. According to the morphological characteristics of the colony in the blood plate and its β- hemolytic ring, strains were further identified as GAS with Lancefield group specific antisera. Genomic DNA was obtained from freshly grown *S. pyogenes* using a Chelex-based DNA extraction kit for genetic analysis, according to the instructions of the DNA extraction kit (Beijing Sangong Bioengineering Co.). The *emm* sequence types were determined using a previously reported protocol (https://www2.cdc.gov/vaccines/biotech/strepblast.asp). Amplicons were sequenced by Guangzhou BGI Genomics Co. Ltd., and *emm* type was determined based on the sequence identity (>95%) of the first 180 bp of the *emm* gene between the tested sequence and the reference *emm* gene.

## Results

### Clinical presentation

The three pediatric cases included in this retrospective analysis included two male patients and one female patient, between the ages of 2 and 7 years. All three children presented with inflammatory manifestations such as localized skin erythema, localized pain, increased local skin temperature, visible pus on puncture and identification of *S. pyogenes* by bacterial culture, confirming the diagnosis of septic arthritis. Details are shown in **Table 1**.

**Table 1:** Clinical data of three pediatric cases of septic arthritis caused by *Streptococcus pyogenes*.

Case	Diagnosis	Chief complaint	WBC (×10^9^/mL)	CRP (mg/L)	ESR (mm/h)	PCT (ng/mL)	Pathology	Culture results	Major antibiotic treatment
**Case 1** Girl, 2y7m	Left suppurative ankle arthritis, sepsis, left fibula distal osteitis	Left foot and ankle swelling and pain for 3 days, accompanied by fever for >1 day	31.81	67	116	3.53	(Left ankle capsule) Fibroadipose tissue, collagen fiber hyperplasia, visible infiltration of lymphocytes, monocytes and some neutrophils	Septic culture: *S. pyogenes*	Vancomycin 4 days, amoxicillin+ sulbactam 22 days
**Case 2** Boy, 7y2m	Left purulent hip arthritis, sepsis	Left lower limb pain and limp for 12 h	32.02	Normal	134	Not measured	Not submitted for inspection	Septic culture: *S. pyogenes*	Cefuroxime for 1 day, desmethylvancomycin for 4 days vancomycin for 33 days, and amoxicillin+ sulbactam for 10 days
**Case 3** Girl, 7y11m	Right purulent hip arthritis, right femur bone, femoral neck osteomyelitis	Right hip pain, limited activity with fever for 2 days	27.68	147.7	113	0.06	(Right hip necrosis tissue) No coated epithelium on the tissue surface, interstitial collagen fibers showed glasslike changes, visible more neutrophil infiltration, vasodilation and congestion, in line with acute suppurative inflammation changes	Septic culture: *S. pyogenes* Blood culture: *S. pyogenes* (penicillin, ceefotaxime, linazolamide, levooxygen, chloramphenicol, ceftriaxone, vancomycin sensitive; clindamycin, erythromycin, tetracycline resistant)	Amoxicillin+ sulbactam for 24 days

WBC, white blood cell; CRP, C-reactive protein; ESR, erythrocyte sedimentation rate; PCT, procalcitonin.

### Laboratory findings

At the first visit, the peripheral WBC count was significantly elevated in all three cases [(27.68–32.02)×10^9^/mL], as was the ESR for all three cases (113–134 mm/h). The CRP level was significantly elevated in two cases (67 mg/L and 147.7 mg/L) and normal in one case. The procalcitonin level was normal in one case, mildly elevated in one case, and not measured in 1 case. Joint X-ray and ultrasound showed local soft tissue swelling and fluid accumulation in the joint cavity in all three cases. For all three cases, treatment included incision and drainage, and pathological examination indicated septic inflammation. Subsequent microbiological culture of the puncture fluid indicated the growth of *S. pyogenes*, and for one patient (case 3), blood culture also identified infection by *S. pyogenes* strains isolated from case 1 and case 2 were *emm*1/ST28 genotype, and case 3 was *emm*12/ST36 genotype. The details are shown in **Table 1**.

### Treatment and follow-up

All three children also received treatment with intravenous antibiotics. Patient 1 was treated with vancomycin intravenously upon initial presentation, and the antibiotic was changed to amoxicillin sodium + sulbactam delivered intravenously after the results of joint fluid culture were obtained. Patient 2 was treated with Cefuroxime for 1 day, desmethylvancomycin for 4 days, vancomycin intravenously for 33 days and then both vancomycin and amoxicillin sodium + sulbactam intravenously for 10 days. Patient 3 was treated with amoxicillin sodium + sulbactam intravenously for 24 days. All three cases showed improvement and were followed up by telephone. No sequelae such as nephritis, rheumatic fever or rheumatic heart disease were reported. Details are shown in **Table 1**.

## Discussion

Septic arthritis is a rapidly progressive, highly destructive and life-threatening joint disease. A study in the UK found that the incidence of septic arthritis increased from 5.5/100,000 in 1998 to 7.8/100,000 in 2013 (Rutherford et al., 2016). According to the literature, the most predominant pathogen causing sepsis in joints is *Staphylococcus*, followed by *Streptococcus pneumoniae* and *Klebsiella*, but also *Salmonella spp*, *Haemophilus influenzae* type b, and group B streptococci (Wang et al., 2003; Frazee et al., 2009; Rutherford et al., 2016; Xu et al., 2016; Dernoncourt et al., 2019; van den Boom et al., 2021; Yagupsky, 2021). *S. pyogenes* can also cause septic arthritis and is easily overlooked. A case of septic arthritis caused by *S. pyogenes* infection was first reported in 1984, and in that case, a serious infection caused by *S. pyogenes* occurred in a nursing home patient and resulted in sepsis, necrotizing fasciitis, septic arthritis, and cellulitis (Ruben et al., 1984). Septic arthritis after toxic shock syndrome caused by *S. pyogenes* was reported in 1995 (González-Ruiz and Ridgway, 1995). *S. pyogenes* infection causing multifocal septic arthritis has also been reported (Marti and Anton, 2007). In contrast, a 10-year case summary in Hong Kong found that in 31 cases of septic arthritis in children, the predominant causative organism was *S. aureus* (42%), followed by *S. pyogenes* (23%) (Kuong et al., 2012). In another study in France, 1 of 26 cases of diffuse infection in infants younger than 3 months of age was septic arthritis due to *S. pyogenes* infection (Germont et al., 2020). Another study in France isolated bacteria from 377 children with suspected joint infections, of which *S. pyogenes* accounted for 15% (Brehin et al., 2020). Methicillin-resistant *S. aureus* infection and septic arthritis combined with osteomyelitis have been reported in the literature as risk factors for the development of sequelae (Wang et al., 2003). A study in Spain reported that the invasive diseases caused by *S. pyogenes* in the Spanish pediatric population include septic arthritis/osteomyelitis, and the common types of *S. pyogenes* are *emm*1/ST28, *emm*12/ST36-ST242, (Sanchez-Encinales et al., 2019). In the present pediatric case series, cases 1 and 2 were *emm*1/ST28 and case 3 was *emm*12/ST36, and these results were basically consistent with those of previous studies. Overall, our case serious combined with the literature reports show that *S. pyogenes* cannot be overlooked as a potential cause of septic arthritis.

Previous research has shown that *S. pyogenes* can invade the joint microenvironment, and step in the development of septic arthritis (Le Hello et al., 2009). Volzke et al (Volzke et al., 2020) inoculated mice intravenously with *S. pyogenes* and observed septic arthritis 3~20 days after infection with increased levels of interleukin (IL)-1β and IL-6 in the joints along with an increased amount of nuclear factor (NF)-κB receptor activator ligand (RANKL), which is a key cytokine for osteoclast formation. *S. pyogenes* infection can increase RANKL expression by increasing the production of activators to induce infectious arthritis.

Septic arthritis occurs in lower limb joints in 90% of cases, with the hip being the most commonly involved, followed by the knee (Wang et al., 2003; Xu et al., 2016). Sternoclavicular joint involvement has also been reported (Dhekane et al., 2020; Kwon et al., 2020). Joint pain (81%) is the most common presentation, followed by fever, swelling and limitation of movement, and 89% of patients show an increased ESR (≥20 mm/h) (Wang et al., 2003). The age at onset of septic arthritis due to *S. pyogenes* varies, with reports of onset in neonates and small infants less than 3 months of age (Umadevi et al., 2013; Germont et al., 2020). The age of onset in our pediatric case series ranged from 2–7 years. A study in Hong Kong reported that only 52% of 31 children with septic arthritis had a temperature below 38.5°C on admission; i.e., nearly half of their patients had a temperature above 38.5°C on admission. Additionally, 71% of their patients had a WBC count below 12×10^9^/L, and the rate of positive blood culture was not high (negative in 74% of cases) (Kuong et al., 2012). In the present case series, all three children presented with a significantly elevated WBC count (30×10^9^/L or more) and a significantly increased ESR. The CRP level was also significantly increased in two cases, while the procalcitonin level was mildly increased in only one case. Previous studies have suggested that procalcitonin is more sensitive than ESR and CRP level for the diagnosis of septic arthritis, and that the combination of these three indicators is beneficial for improving diagnostic sensitivity and specificity (Wang et al., 2014; Wei et al., 2015). In contrast, Chen (Chen et al., 2013) found that the CRP level is more sensitive than procalcitonin for the identification of local bacterial infection. The results in our pediatric case series are more consistent with the findings of Chen et al (Chen et al., 2013).

The standard treatment for septic arthritis in children is arthrocentesis combined with antibacterial drug therapy (Kwon et al., 2020). Studies have shown that adequate use of antimicrobial drugs and a single arthrocentesis is sufficient to treat septic arthritis in most pediatric cases, regardless of the infecting agent or site of infection, as long as the clinical response is good (Peltola et al., 2009). Additional research had indicated that early antibiotic treatment, incision and drainage, and combined non-pharmacological treatments such as drainage and early physiotherapy should be given (Couderc et al., 2020). More recent studies have suggested that targeted synovial cell therapy may be a promising treatment for septic arthritis (Volzke et al., 2020). Penicillin has been widely used worldwide for many years, and so far, no penicillin-resistant strains of *S. pyogenes* have been identified. The reasons for this are not yet known. In recent years, increased minimum inhibitory concentration (MIC) values of penicillin have been reported, and penicillin-nonsusceptible *S. pyogenes* strains have emerged. In 2006, Capoor et al. (Capoor et al., 2006) reported *S. pyogenes* with elevated MIC values to penicillin (0.19~0.25 µg/mL), and *S. pyogenes* with elevated MIC values to penicillin (0.25~0.75 µg/mL) were also found in Mexico (Ogawa et al., 2011). Additionally, several resistance surveillance networks in China have reported *S. pyogenes* “resistance” to β-lactam antimicrobials, but we confirmed that these strains are not truly resistant, and whether they carry a mutated gene for penicillin-binding protein 2X (pbp2x) needs to be confirmed by further studies (Vannice et al., 2020; Musser et al., 2020; Yu et al., 2020). In the present case series, *S. pyogenes* was cultured from the joint cavity pus of all three children, and they were considered sensitive to β-lactam antibiotics based on the outcomes in these cases. No antibiotic susceptibility testing was performed. However, in case 3, *S. pyogenes* was cultured from the blood, and antibiotic susceptibility testing suggested sensitive to β-lactam antibiotics such as penicillin and cephalosporin. For this case, amoxicillin sodium + sulbactam was selected and provided satisfactory treatment. Therefore, in clinical practice, once pus culture identifies *S. pyogenes*, the choice of β-lactam antibiotics is sufficient, and prompt step-down treatment should be given, as it is not advisable to continue advanced antibiotic therapy. In case 1 of our series, after the pus culture result was clear, vancomycin was promptly changed to amoxicillin sodium + sulbactam with good effect, while in case 2, vancomycin combined with β-lactams treatment was considered to be related to the doctor’s insufficient knowledge of *S. pyogenes*. In case 3, intravenous infusion of amoxicillin sodium + sulbactam was administered from the time of admission with good effect.

## Conclusion

In conclusion, antimicrobial agents commonly used to treat *S. pyogenes* infections are highly active against clinical strains.*S. pyogenes* is an important pathogen causing septic arthritis, and WBC count, ESR, and CRP level are sensitive indicators for the diagnosis of septic arthritis. If *S. pyogenes* infection is confirmed upon culture of pus drained from an infection joint, β-lactam antibacterial therapy with antibiotics such as penicillin or cephalosporin can be selected for treatment.

## Data availability statement

The original contributions presented in the study are included in the article/supplementary material. Further inquiries can be directed to the corresponding authors.

## Ethics statement

The studies involving human participants were reviewed and approved by Shenzhen Children’s Hospital. Written informed consent to participate in this study was provided by the participants’ legal guardian/next of kin.

## Author contributions

WW and YY contributed to conception, design, and administrative support. DY, QL, WG, DG,YC, and YZ provided study materials and patients. DY contributed to the collection and assembly of data, data analysis, interpretation and the manuscript writing. All authors contributed to the article and approved the submitted version.
